# Model of Fission Yeast Cell Shape Driven by Membrane-Bound Growth Factors and the Cytoskeleton

**DOI:** 10.1371/journal.pcbi.1003287

**Published:** 2013-10-17

**Authors:** Tyler Drake, Dimitrios Vavylonis

**Affiliations:** Department of Physics, Lehigh University, Bethlehem, Pennsylvania, United States of America; North Carolina State University, United States of America

## Abstract

Fission yeast serves as a model for how cellular polarization machinery consisting of signaling molecules and the actin and microtubule cytoskeleton regulates cell shape. In this work, we develop mathematical models to investigate how these cells maintain a tubular shape of approximately constant diameter. Many studies identify active Cdc42, found in a cap at the inner membrane of growing cell tips, as an important regulator of local cell wall remodeling, likely through control of exocyst tethering and the targeting of other polarity-enhancing structures. First, we show that a computational model with Cdc42-dependent local cell wall remodeling under turgor pressure predicts a relationship between spatial extent of growth signal and cell diameter that is in agreement with prior experiments. Second, we model the consequences of feedback between cell shape and distribution of Cdc42 growth signal at cell tips. We show that stability of cell diameter over successive cell divisions places restrictions on their mutual dependence. We argue that simple models where the spatial extent of the tip growth signal relies solely on geometrical alignment of confined microtubules might lead to unstable width regulation. Third, we study a computational model that combines a growth signal distributed over a characteristic length scale (as, for example, by a reaction-diffusion mechanism) with an axis-sensing microtubules system that places landmarks at positions where microtubule tips touch the cortex. A two-dimensional implementation of this model leads to stable cell diameter for a wide range of parameters. Changes to the parameters of this model reproduce straight, bent, and bulged cell shapes, and we discuss how this model is consistent with other observed cell shapes in mutants. Our work provides an initial quantitative framework for understanding the regulation of cell shape in fission yeast, and a scaffold for understanding this process on a more molecular level in the future.

## Author Summary

Fission yeast is a rod-shaped organism that is studied, in part, as a model for how cells develop and regulate their shape. Despite extensive work identifying effects of genetic mutations and pharmacological treatments on the shape of these cells, there is a lack of mathematical and computational models examining how internal cell signals and the cytoskeleton organize to remodel the cell wall, direct growth at cell tips, and maintain tubular shape. In this work we describe how the spatial distribution of regulatory protein signal at growing cell tips relates to cell diameter. Further, we describe the consequences of this signal depending on the shape of the cell, namely its length and diameter. Finally, we propose a computational model for understanding growth and shape that includes an axis-sensing microtubule system, landmarks delivered to cell tips along those microtubules, and a growth zone signal that moves around but is attracted to the landmarks. This picture explains a large number of reported abnormal shapes in terms of only a few modular components.

## Introduction

**Figure 1:**
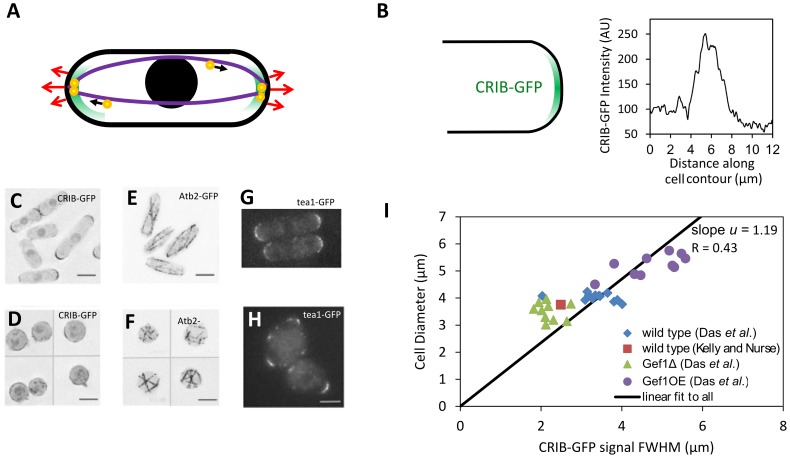
Fission yeast cell shape and regulation by the Cdc42 and microtubule systems. **A.** Schematic of fission yeast. Cell outline and nucleus in black, red arrows indicate outward cell wall expansion during bipolar growth, green represents growth-factor Cdc42 signal, purple shows microtubules aligned to the long axis, orange-and-yellow circles are protein-carrying vesicles delivered along microtubules that mark the tips. **B.** CRIB-GFP (a marker for active Cdc42 [29]) localizes at cell tips. Plot shows CRIB-GFP intensity measured along the contour of a cell tip in Fig. 1A of [15]. **C.** CRIB-GFP fluorescence in control cells. **D.** CRIB-GFP after enzymatic digestion of the cell wall that causes cell rounding. CRIB-GFP appears to accumulate in patches along the cell surface. **E.** Atb2-GFP fluorescence shows microtubules in control cells. In elongated cells microtubules align along the long axis of the cell. **F.** Atb2-GFP in rounded cells (after enzymatic digestion of the cell wall as in **D**) shows microtubules with random orientations. **G.** Tea1-GFP, delivered to cell tips by microtubules shows tip-marker location in wild type cells. **H.** Tea1-GFP fluorescence in nearly-round *sla2Δ* cells reveals misplaced tip markers. (C–F: reproduced from [16]; G, H: reproduced with permission from the Journal of Cell Science [57]). **I.** Cell diameter versus CRIB-GFP signal full-width half-max (measured as in 1B) for wild-type cells and cells with modulated levels of Gef1, a Cdc42 activator. The fit is constrained to go through the origin in order to match the form of the model that predicts the ratio of cell diameter to FWHM. A fit not constrained through the origin gives slope = .57, and intercept 2.15 µm (R = 0.86).

Many cells such as fungal hyphae, pollen tubes and some bacteria grow from their tips by remodeling their cell wall [1]–[3]. Fission yeast (*S. pombe*) also grow this way and, as a well-studied model organism, are good for understanding tip growth and, more generally, the mechanisms of acquisition of cell shape [4]–[16]. Wild type fission yeast cells have a cylindrical shape and maintain a diameter of about 3.4 µm and double in length from approximately 7.5 microns to 15 microns during their life cycle (see Fig. 1A). Many fission yeast shape mutants have been identified [7]. Common shape mutants include round cells [7], [8], cells with wider or thinner diameter [9]–[11], and branched cells [7], [12].

Fission yeast and other eukaryotic tip-growing cells use Rho GTPase signaling and the cytoskeleton to maintain polarized growth [6], [13]. Prior work identified two core modules that regulate distinct aspects of fission yeast shape [6], [14] (see Fig. 1A): (i) The small Rho GTPase signaling protein Cdc42 and its associated proteins establish a system that influences the width of the growth zone [11], [15], [16]. Along with its activators and inhibitors, guanine nucleotide exchange factors (GEFs) and GTPase-activating proteins (GAPs) and actin-mediated transport, the Cdc42 system contributes to the formation and upkeep of a growth zone with characteristic width [11] (see Fig. 1B, C, D). By accumulating at the cell tips, active Cdc42 defines an area where vesicle delivery, exocytosis [17], and cell wall remodeling occurs by delivery of cell wall synthases [18], [19]. (ii) Microtubules align along the long axis of the cells and deliver landmark proteins to the tips, thus defining the tip region and maintain a straight central axis [4], [20], [21] (see Fig. 1E, F). Microtubules provide a directed track for kinesin-based delivery of +TIP proteins to the cell tip, such as Tea1 [4] (see Fig. 1G, H). The microtubule system detects shape and marks the cell tips even in mutant cells that lack the ability to direct growth but have been confined to narrow microchannels, and fails to mark the cell tips and instead marks regions on the side of the cell tip if physical restrictions force a large shape change [20]–[22].

While a large body of experimental work has identified genetic mutations that result in modified cell morphology, such as polarity and width, there has been little modeling work [23] to identify which physical features are required for maintaining cell shape in fission yeast. Cell-scale features such as polarity and width arise from protein-scale cell-wall remodeling and expansion events. Signaling proteins, because they function through short-range interactions, likely operate on a molecular level as well. The specific mechanisms of growth are likely very complex to allow modeling at a molecular level at present; for example, Cdc42 regulates at least two parallel growth pathways [17]. Because of the large separation of scales, however, we anticipate that the cell relies on a modular mechanism that could be approximately described by a coarse-grained model that incorporates the main features of the system. In this modeling study, we explore how the two modules, one based on Cdc42 and another based on microtubules, act in concert to achieve robust regulation—and even recovery—of shape.

We first describe a model for how cell diameter depends on the distribution of a steady Cdc42 signal on the cell tip. Secondly, we study the implications of having a distribution of Cdc42 at the tip that depends on cell shape and show that stability of cell diameter conditions constrain the possible mechanisms for shape-dependent signal. Finally, we show that a model combining a Cdc42 signal distributed over a characteristic length-scale (as could be generated by a reaction-diffusion process), Cdc42-signal-dependent cell expansion, and microtubule-dependent detection of the long axis of the cell can generate cells with stable diameter and cells with shapes of known mutants. Given what is known about the mechanistic roles of the missing or affected proteins, these results are consistent with the proposed mechanisms of shape regulation.

## Results

### Model for Remodeling under Turgor: From Membrane-Bound Growth Factor Distribution to Cell Shape

Calcofluor staining that marks new cell wall, reveals that new material during vegetative growth is incorporated at cell tips [24], where Cdc42 accumulates. Two studies [11], [15] using a fluorescent marker for active Cdc42 found an approximately Gaussian intensity profile along the meriodonal contour with a maximum intensity at the cell tip (Fig. 1B). In one of these studies, active Cdc42 distribution in cells that overexpress or lack Cdc42 activator Gef1 show that cell diameter correlates with the width of the active Cdc42 signal profile [15] (Fig. 1I). Since Cdc42 targets actin cables to cell tips [17] and also helps the cell target the exocyst using PIP2 independently of the cytoskeleton [17], and because these two parallel pathways are thought to be responsible for bringing the relevant cell-wall synthases such as Bgs1 and Bgs4 [18], [19] to the tips for polarized growth, it is likely that the rate of cell wall expansion depends on the local concentration of active Cdc42. Newly deposited cell wall material will deform under turgor pressure: turgor pressure likely deforms the cell wall within the range of its elastic response, because even cells bent by confinement in stiff microchambers sometimes recover their shape within seconds [22].

**Figure 2:**
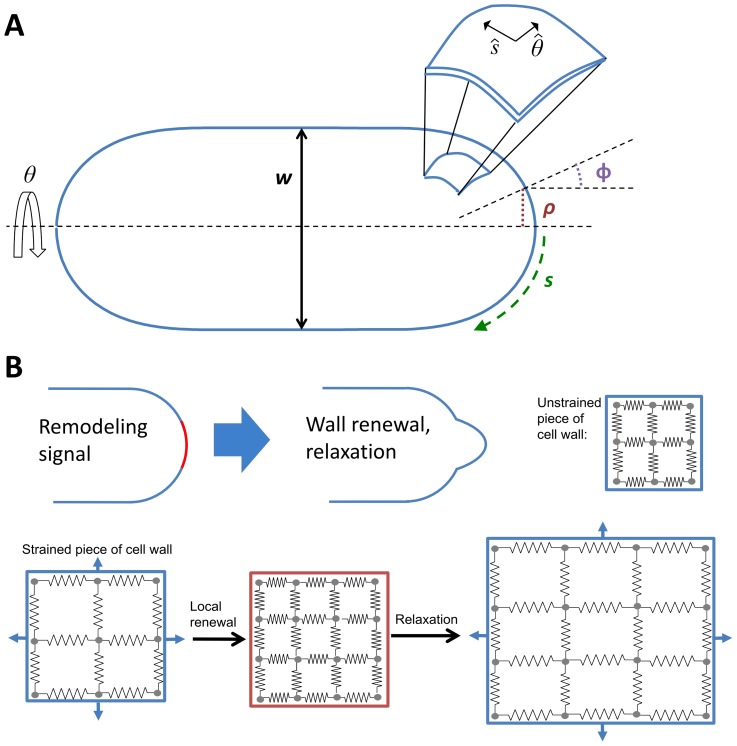
Coordinate axis and model of elastic cell wall remodeled under turgor pressure. **A.** Axisymmetric cell, meriodonal distance *s*, distance from axis of symmetry *ρ*. Angle *φ* is the angle between axis of symmetry and the normal to cell surface. Angle *θ* is measured around the axis of symmetry. Enlarged part of cell wall shows unit vectors 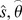 along *s* and *θ*. **B.** Illustration of cell wall remodeling model. Illustration of how remodeling signal causes part of cell wall under tension to remodel and relax to new shape. Bottom: replacement of strained cell wall by unstrained material followed by stretching of cell wall under turgor pressure. In the model the two processes happen simultaneously.

Can a model of cell growth where the cell wall is represented as an elastic boundary (the peptidoglycan matrix) under turgor pressure being remodeled at growth zones that polarized cells place at the tips predict the correct cell diameter? What is the relationship between the size and shape of the growth-zone signal and cell shape and diameter? Taking into account the above experimental observations, we developed a model of cell growth in which an elastic boundary under turgor pressure is remodeled at growth zones marked by Cdc42 at the tips. This model is a modified version of a model by Dumais, *et al.* [25] (see Discussion for comparison to other models of tip shape). Specifically we assume that the process of wall expansion can be described by the replacement of cell wall material strained by osmotic pressure by unstrained material. Local cell wall remodeling occurs at a rate proportional to the local concentration of a growth factor Λ(*s*), where *s* is distance from cell tip, see Fig. 2. Function Λ(*s*) represents the *s*-dependent concentration of Cdc42 and other proteins that contribute to wall remodeling. We assume that the material delivered according to Λ(s) is able to maintain a wall of constant thickness around the cell, through local cell wall digestion and synthesis processes. In this section we assume that the growth-zone signal Λ(*s*) remains constant during cell growth but in the next section we will consider the effect of the signal being also dependent on cell size.

First, we calculate the stresses, *σ*_s_ and *σ_θ_*, necessary to balance turgor pressure *P* for an arbitrary simple axisymmetric shape where the position of a piece of cell wall is described by the distance to cell tip, *s*, and angle *θ* (Fig. 2A). This depends on cell wall thickness, *δ*, and the principal curvatures, *κ*_s_ and *κ*_θ_ [26]:
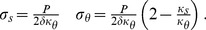
(1)For compactness we will occasionally write *σ* instead of *σ*(*s*), and so on. The balance of stresses in Eq. (1) assumes that growth through wall remodeling occurs slowly compared to the timescale required to establish mechanical equilibrium (hence the cell wall does not expand unless remodeled). From the elastic stress–strain relationship, which includes the Young's modulus, *E*, and the Poisson ratio, *ν*, the corresponding strains are:
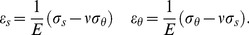
(2)We assume that during remodeling strained material is replaced by unstrained material with thickness held constant, corresponding to the wall expanding under turgor pressure in proportion to local strain (Fig. 2B). The signal Λ(s) directs remodeling, and the cell wall expansion rates *ξ_s_* and *ξ_θ_* are the product of the strain and the remodeling rate set by the signal:
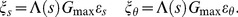
(3)Here, we assume that Λ(0) = 1 and constant *G*_max_ is the remodeling rate at the cell tip. Geometrical considerations relate the expansion rate to the velocities along the normal and tangential directions of the surface, *v_n_* and *v_t_*, of a piece of the cell wall with coordinates *s* and *θ* [26]:
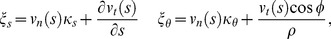
(4)where *φ* is the angle between the normal vector and the long axis of the cell and *ρ* is the distance to the long axis, see Fig. 2A. The velocities here are with respect to a frame of reference where 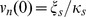, meaning the motion at that tip is due to only local expansion.

**Figure 3:**
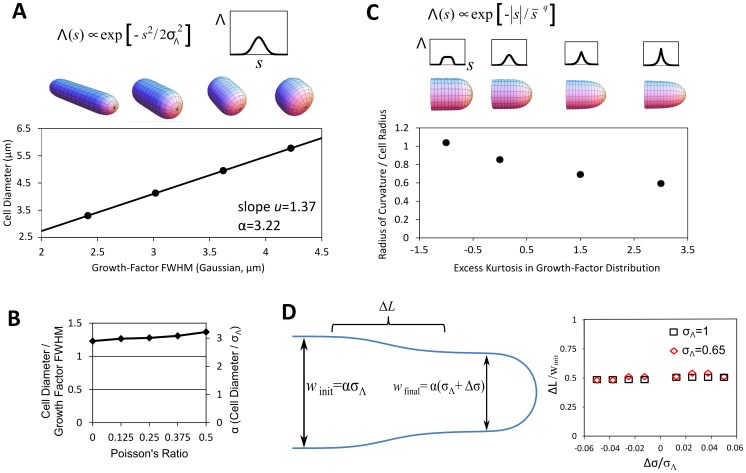
Model for remodeling induced by growth factor yields several predictions. **A.** For a Gaussian growth-factor signal, cell diameter is proportional to signal width as described in the main text. **B.** Effect of changing Poisson's ratio of material inserted on the slope in A. **C.** Using for input an exponential power distribution shows that a pointier or blunter signal gives a pointier or blunter cell. Excess kurtosis measures the peakedness of the distribution. Parameters *q* and 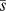 are found numerically to match values of excess kurtosis while keeping the standard deviation constant. Plot shows ratio of tip radius of curvature to cell radius. We note that for each value of excess kurtosis, the dependence of diameter on the standard deviation of the signal is described by a different value of α (not shown). **D.** Length of the region of a transition between two widths after an instantaneous change in the width σ_Λ_ of a Gaussian growth signal by Δσ. Illustrative simulated cell outline showing the cell border (blue), region of a transition with length Δ*L*, and cell diameters *w*_init_ and *w*_final_ of two regions of the growing tip. The size of the transition region, measured as the length of the region spanning the middle two quartiles in width, is proportional to cell width and remains nearly constant as Δσ increases or decreases.

We solved Equations (1)–(4) numerically (see Methods) to calculate steady-state tip shape as a function of growth-factor signal Λ(*s*), see Fig. 3. Osmotic pressure *P*, Young's Modulus *E*, and the thickness of wall *δ* combine to form *G_max_ P/Eδ*, a constant that affects the speed of expansion but not the steady-state shape (see Methods section). Therefore, the only factors that determine the change of shape are the geometrical properties of the contour and the signal Λ(*s*). Simulation results starting from a variety of initial contours reach the same steady-state tip shape, showing that the final calculated shape depends only on Λ(*s*).

We found that the diameter of the steady state cylindrical projection increases linearly with the full-width half-max (FWHM) of a Gaussian signal (Fig. 3A). Of course this must be the case because the width of the signal is the only length scale in the model, but the model predicts the ratio *u* of the cell diameter to the FWHM of the signal ranges from 1.23 to 1.37 as the Poisson ratio of the material inserted ranges from 0 to 0.5, see Fig. 3B. Equivalently, the ratio of cell diameter to the standard deviation of the signal 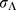, which we call α = 2.35 *u*, ranges from 2.89 to 3.22. The calculated value of *u*, which is not the result of a fit, is close to 1.19, the value of the slope in the experiments of cell diameter versus CRIB-GFP signal FWHM in Fig. 1I. We also tested how the shape of the growth projection depends on the form of Λ(*s*) by using the exponential power distribution (Fig. 3C). This reveals what we might naively expect: a blunter or pointier signal gives a blunter or pointier cell. In order words, the precise shape of the growth projection changes in the same way the signal does. This would be consistent with observations in budding yeast, where it has been speculated that a sharper Cdc42 profile contributes to the pointier shape of the shmoo as compared to a rounded bud [27]. We do not know of a genetic alteration that produces a drastically non-Gaussian cortical active-Cdc42 profile but recent results suggest that cells lacking Mid1 may be able to grow pointier tips under some conditions [28], which might occur by sharpening the Cdc42 profile through Pom1-dependent regulation by Rga4 [29]. Our results also suggest testing if the ice-cream cone shape observed in some mutants [30] is due to a non-Gaussian Cdc42 profile or else due to a Gaussian Cdc42 profile with cell-length-dependent width.

We also performed simulations to study how quickly the cell diameter equilibrates after a change in the growth signal. Fig. 3D shows simulations that start from a steady state protrusion generated by a Gaussian signal; the signal width is instantaneously changed by Δσ and the shape is followed over time. We found that the cell diameter equilibrates to the new steady state when the protrusion extends by approximately half a cell diameter, irrespective of the magnitude or sign of Δσ.

Integrating the model (see Methods section ‘Evolution of tip shape as function of growth-factor signal Λ(s)’), and factoring out physical constants (leaving a dimensionless integral) uncovers a relationship between growth velocity and the parameters of the model:
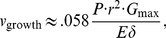
(5)where *r* is cell radius and the numerical prefactor depends on the shape of Λ(*s*) (here Gaussian) and on the Poisson's ratio of the material being inserted. Here we use a Poisson's ratio of 0.5; the value of the prefactor increases by 96% as Poisson's ratio is decreased to zero because the strain approximately doubles (see Eq. (2) where *σ*_s_ and *σ_θ_* are typically of same order of magnitude). Thus, growth velocity scales linearly with turgor pressure. This linear relationship agrees with the experimental findings in [22], where a change in turgor pressure was simulated by confining cells in elastic chambers and regulating osmolarity with sorbitol [22]. Using 1.6 microns for the cell radius, a turgor pressure of .85 MPa [22], a cell-wall thickness of 200 nm [31], and a Young's modulus of 101 MPa [22], along with a velocity 2 µm/hr that corresponds to the cell doubling length in its cycle with a constant velocity, we estimate for *G*_max_∼0.33 sec^−1^ (see Table S1 for physical parameters related to the model in this section). While this is only a rough estimate, this number differs by approximately one order of magnitude from 0.022 sec^−1^, an independent estimate of the rate membrane is internalized by endocytosis found by multiplying 25 actin patches per tip [32] by the area of a 300 nm vesicle [33], dividing by the area of a 4.5 µm-wide growth zone [15], and dividing by the 20-second lifetime of an actin patch [34]. Perhaps the discrepancy exists because remodeling of cell wall by cell wall synthases and hydrolases happens at a faster rate compared to the rate of membrane delivery or removal at cell tips.

### Shape-Dependent Growth Signal and Maintenance of Cell Diameter

The model of the previous section showed that a steady signal for cell wall growth distributed according to the measured active Cdc42 distribution at cell tips can generate a cylindrical extension with a diameter approximately equal to the measured diameter of fission yeast cells. However the spatial distribution of the signal that determines cell growth also depends on the cell shape generated by the signal. Moreover, fission yeast doubles in volume before division and the distribution of growth signal around the cell tip may vary during the cell cycle. For example, the Cdc42 signal changes from monopolar to bipolar distributions; as cells grow the signal oscillates and fluctuates, and may also change in width [15]. How can the cell maintain a stable cell diameter despite these mutual dependencies? In this section we explore the importance of the mutual dependence between signal and shape.

Since the period of Cdc42 oscillations [15], ∼5 min, is much shorter than the doubling time, we expect that using the time-averaged Cdc42 profile along cell tips would be a good approximation for Λ(*s*). We anticipate a bigger effect is the dependence of Λ on cell shape. The main feature of Λ that determines the width of the growing projection is its standard deviation, 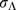 (Fig. 3A). Since wild type cells are approximately spherocylindrical, we approximate the dependence of signal on cell shape by function 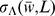, where 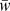 is the average cell diameter along the cell length excluding the tip cap regions and *L* is cell length. Here we allow the cell diameter to vary slightly along the cell axis but assume that the average diameter 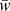 and cell length *L* are the features of shape that determine 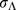 (as long as cells remain approximately spherocylindrical).

The diameter of the growing portion of the cell changes according to 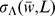. This causes the average cell diameter to change with length, making 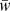 a function of *L*. This interplay between the diameter of the cell and the extent of a signal for remodeling can be described by:
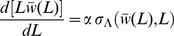
(6)In Eq. (6) we assume the diameter of the growing portion depends only on the current average dimensions of the cell (here we neglect the transient ΔL associated with equilibration of the diameter of the growing portion to a change in 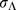 shown in Fig. 3D). The product inside the derivative of the left-hand side can be understood as the area of a length-wise cross-section of the cell. The derivative of this product with respect to changes in length is equated to the signal-dependent diameter of a growing tip. Here α is a constant given by the model of the preceding section that ranges between 2.89 and 3.22 for a Gaussian Λ (see Fig. 3A, B). Eq. (6) is valid for growth from just one tip or from both tips (as long as both tips have the same growth signal distribution). Starting with an initial length 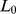 after cell division and initial average diameter 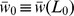, integration of Eq. (6) gives the average cell diameter 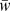 when cell reaches length *L*. To maintain constant cell diameter through repeated cell growth and division, the average cell diameter at division must be equal to the initial diameter 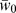. This gives the following requirement:
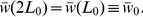
(7)


The diameter of wild type cells does not change significantly throughout the cell cycle, so we can assume that 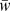 changes by a sufficiently small amount to allow us to perform a linear expansion of 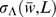 in Eq. (6). Expanding around the initial diameter 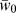, one has:
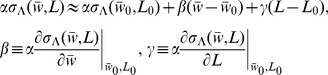
(8)The above equations allow us to calculate the steady state cell diameter at birth, for a given dependence of the growth signal width on cell shape. Substituting Eq. (8) into Eq. (6) and applying the steady state condition, Eq. (7), gives the steady state cell diameter at birth, 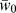:
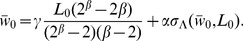
(9)The solution of Eq. (9) for 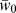 defines a fixed point for the system. Similarly, we find the average cell diameter as function of cell length:
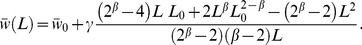
(10)
Eq. (10) shows that the average cell diameter goes through a maximum or minimum as cells grow, depending on the sign of *γ*. Our linear expansion is self-consistent when parameter 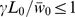, making the term added to 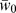 in Eq. (10) a small correction compared to the initial diameter (for *β* = 1, the maximum value of the third term in Eq. (10) is ∼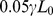). The linear expansion in Eq. (8) works as long as the magnitude of *β* is of order unity, or less, after comparing the magnitudes of the last two terms in Eq. (8).

**Figure 4:**
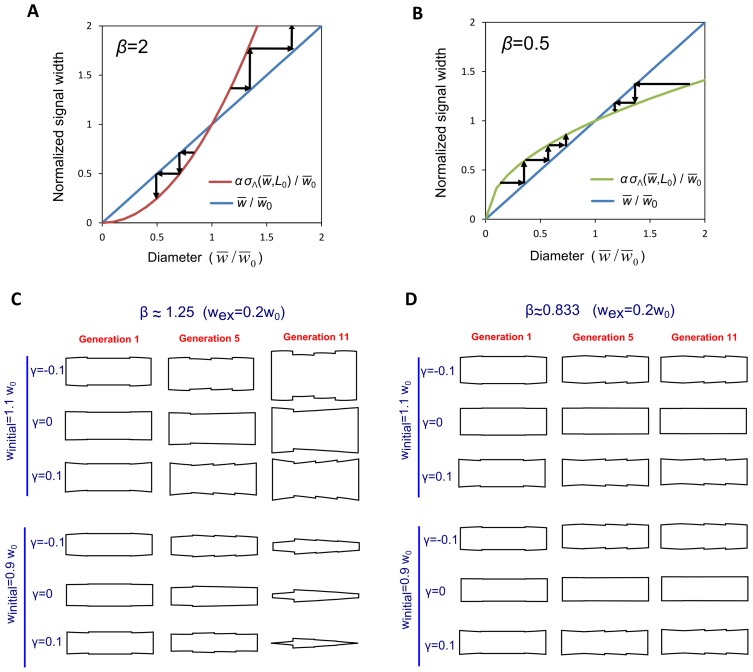
Stable diameter maintenance depends on how growth signal width varies with cell diameter (quantified by the value of *β*, see Eq. (11)). **A.** Unstable case, *β*>1. Plot shows normalized growth signal width 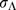 versus cell diameter 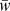, along with the diagonal. The intersection between diagonal and 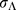 curve determines steady state cell diameter 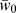, see Eq. (9) (for simplicity here we assume no cell length dependence of 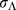, corresponding to *γ* = 0). Small perturbations of the diameter from the value of the fixed point are amplified after successive cell divisions. Arrows show how increased (decreased) diameter leads to wider (narrower) growth signal that in turn causes an increased (decreased) diameter. **B.** Same as A, but for stable case, *β*<1. Perturbations in diameter are corrected, the fixed point is stable and cells maintain constant diameter. **C.** Successive shapes of the cylindrical part of cells generated by model of Eq. (6) demonstrate instability for *β*>1. Cells begin with a rectangular shape and grow from tips according to Eq. (6). We use a signal similar to Eq. (12) that includes a dependence on both average cell width and length: 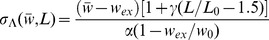. We consider small *γ* such that the steady state diameter is 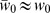 (see Eq. (9)). We used 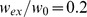, corresponding to 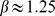 and *L*_0_ = 2.5 w_0_. Parameter *γ* and the perturbed value of the initial width, *w*_initial_, are indicated on figure. Shapes of sample cells in the population are shown before the first, fifth, and eleventh divisions. Cells become wider and wider or thinner and thinner depending on whether *w*_initial_ is smaller or larger than 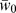. The abrupt changes in width along cell length originate in the abrupt change of growth signal width following cell division. These discontinuities will be smoother than in the model of Eq. (6), smoothed over a distance equal to cell radius, see Fig. 3D **D.** Same as B, but for 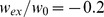, corresponding to 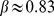. In this case cells start wider or thinner than 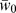 but converge back to the steady state diameter.

We have calculated a fixed point for cell diameter (Eq. (9)), but this point is not necessarily a stable one. Performing linear stability analysis of Eq. (6), and requiring that if 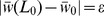 then 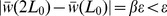 leads to:
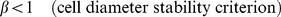
(11)Thus, the signal that determines cell growth expansion may become more widely distributed across the cell tip with increasing cell diameter (*β*>0), but this dependence has to be weak enough, according to Eq. (11). When *β*<1, a small increase in cell diameter away from its steady state value (by a perturbation or random fluctuations, for example) will cause signal expansion; however the cell diameter generated by the modified growth signal will not be as large as the initial perturbation and as a result the cell diameter will eventually return to its steady state value. When *β*>1, small changes in cell diameter get reinforced by the resulting large change in growth signal and thus the cell continues to become wider and wider (or thinner and thinner). This is illustrated in Fig. 4 that shows stable and unstable cell shapes after successive divisions, for various values of *β* and *γ*. .

### Microtubule-Based Distribution of Growth Signal and Stability of Cell Diameter

In fission yeast, the most obvious shape-sensing organelle is the microtubule cytoskeleton (see Fig. 1). During interphase, the part of the cell cycle where the cell elongates, microtubules radiate from three to five organizing centers attached to the nucleus [35]. From these centers, approximately two microtubules usually extend tipward in either direction, undergoing catastrophes and rescues [36], [37], often spending one or two minutes probing the cell-tip region before shrinking away after a catastrophe. Their alignment appears to depend on geometrical confinement within the cell [20], [21]. In addition to centering the nucleus, these microtubules target the delivery of polarity regulators to the cell tips. Microtubules allow motor proteins such as kinesin Tea2 along with plus-tip proteins like Tip1 and Mal3 to deliver cargo such as protein Tea1 to the cell tips [38], [39]. Tea1 forms a complex with Tea4 that also localizes formin and actin-cable-nucleator For3 to cell tips [40].

If a narrow tubular shape helps to focus microtubule tips, and if microtubule-dependent polarity effectors direct growth, can the microtubule system be the main mechanism for maintenance of diameter and rod-like shape of fission-yeast? In other words, is a mechanism where the width of the growth signal is primarily determined by the width of microtubule-based delivery able to maintain cell diameter? Indeed, this has been proposed in at least two experimental studies of fission-yeast shape [20], [21]. The discussion of the preceding section shows that the feasibility of such a mechanism depends on criteria such as Eq. (11) that have not been explored quantitatively.

As a simple, instructive, model of how microtubule-based signal could help control cell diameter, let us approximate the cell as two caps connected by a cylinder of diameter 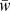. Let us also assume that microtubules deliver cell tip growth proteins approximately uniformly near the cell tip, avoiding or extending past the corner region connecting the caps to the cylindrical body by a distance of size 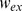. We further assume that the microtubule system can achieve this in a way that signal width 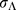 is independent of cell length, i.e. *γ* = 0. One has:
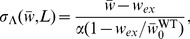
(12)where the denominator is a scaling factor that gives the wild type diameter 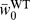 as the fixed point in Eq. (9) and 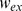 is positive (negative) when the growth zone is smaller (larger) than the cap region. The magnitude of the width of such a growth signal would be consistent with the experimentally-measured Tea1 profile that is approximately Gaussian with standard deviation ∼1 µm [41]. For this dependence of signal on shape, we find, using Eq. (8):
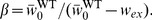
(13)This *β* value is larger than unity for any positive 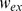. Thus, for positive 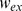 the signal distribution of the simple model of Eq. (12) is an unstable width regulation mechanism. It can be shown that other such simple geometrical models can give values of 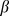 that are smaller than unity, but are generally near unity. Hence, microtubule-only-based models of cell diameter regulation may suffer from instability problems.

**Figure 5:**
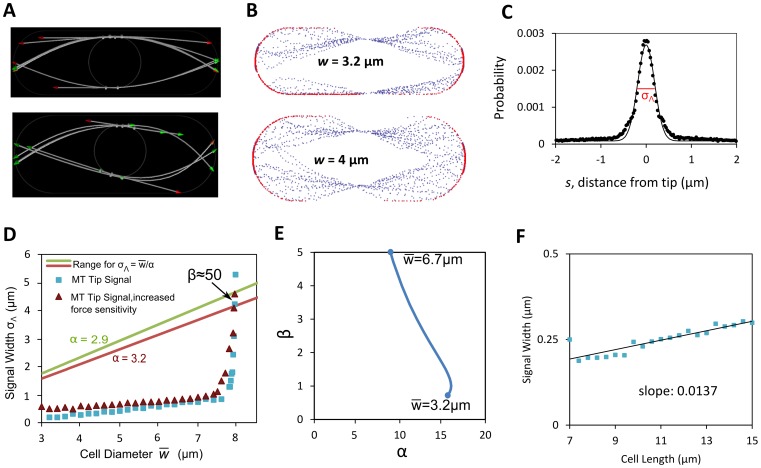
Microtubule-only model of growth signal distribution leads to unstable width regulation. **A.** Snapshots of microtubule distributions using the 2D version of model of Foethke *et al.* [42] for two different cell widths. **B.** Microtubule tip locations (red: touching cell boundary; blue: not touching) extracted from model shown in A. **C.** Time-averaged probability distribution of microtubule tips touching cell boundary as function of distance from cell tip. Continuous curve: Gaussian fit. **D.** Standard deviation of microtubule tip signal from plots as in panel C, versus cell diameter for two parameter sets shown in Table S2. Cell length: 8 µm. The red and green lines show curve 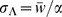 using the upper and lower values for parameter α that depends on the value of Poisson's ratio for inserted material (see Fig. 3B). The intersection between the tip signal width curve and the straight lines is the fixed point that determines the cell diameter, see Eq. (9) (here the value of γ is small, see panel E). The slope of 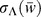 at the intersection determines stability of cell diameter (see Fig. 4). The graph shows a fixed point at the wrong diameter (near 8 µm instead of ∼3.2 µm), and that that fixed point is unstable since *β*>1 at the intersection (similar to Fig. 4A). **E.** *β* versus *α* obtained from signal 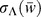 in panel D. Graph was obtained by first fitting 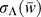 to an analytical function. We then varied *α* to find the intersection of the straight line in D with the 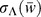 curve. The value of *β* was calculated at the intersection using Eq. (8) and the analytic form of the derivative. The value of 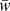 at the interestion is shown at two points of the curve. Note that stability (*β*<1) occurs only for values of *α* above 15, while the range found for *α* found in Fig. 3B is 2.89 to 3.22. **F** Standard deviation of microtubule tip signal varies slightly with cell length. Cell diameter = 3.2 µm. Line shows least-squares linear fit with slope 0.0137.

Microtubule bending and buckling, which is observed in fission yeast [42], could focus the microtubules near the cell tip and could reduce the dependence of tip growth factor delivery width on cell diameter compared to the model of Eq. (12), thus providing a stable width regulation mechanism. To explore this possibility, we employed a detailed computational model of microtubules proposed by Foethke and others [42], which treats the microtubules as growing and shrinking flexible rods attached to a spherical nucleus in a viscous fluid by drifting springs (see Fig. 5A). The persistence length of microtubules in the simulations was 7.3 mm; while several orders of magnitude longer than the cell length, the pN forces generated by polymerization are large enough to bend and buckle the microtubules that grow against a cell tip [42]. We used the two-dimensional version of their model (available at www.cytosim.org) that allows extracting locations of positions of microtubule tips. We expect the 2D version to give similar results to the full 3D model, since each microtubule lies approximately on a 2D plane. Microtubule catastrophe rates, in that model, increase with both the length of the microtubule and the force on the tip. Using that model, we changed the diameter and length of the two-dimensional confining cell and tracked the coordinates of many microtubule tips (see Table S2 for model parameters). This gives a profile of where the microtubule tips touch the cell boundary during interphase as a function of cell diameter (see Fig. 5B, C). Snapshots of simulations in Fig. 5A show configurations of microtubules and the focusing effect of buckling.

As an approximation for the microtubule-based growth signal width 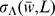 derived from the simulations of Fig. 5A, we examined a model in which the growth factor distribution across the cell tip is equal to the distribution of the likelihood of microtubule tip contact per unit area (see Fig. 5B). Such a model assumes that a localized growth factor signal is delivered in proportion to the time-averaged density of microtubule tips touching the cell membrane. Repeated simulations of microtubule dynamics give a frequency distribution for the location of microtubule tips as a function of the meridional distance (Fig. 5C). This probability density function is fitted to a Gaussian distribution and the standard-deviation fit parameter 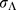 as function of cell diameter 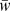 and length is shown in Figs. 5D and F. (Note: Conversion of the distribution of Fig. 5C to the corresponding 3D distribution before extracting parameter 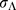 does not change the following conclusions).

The signal 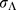 of the model described in the preceding paragraph generates the wrong cell diameter, which is also unstable. Plot of signal width 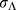 as a function of cell length in Fig. 5F shows a weak length dependence, *γ*≈0.04. The dependence of 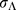 on cell diameter is approximately linear for cell diameters smaller than 7 µm, for a cell length 7 µm (Fig. 5D). As the diameter becomes comparable to cell length however, a sharp unfocusing transition occurs and 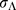 increases rapidly (spike in Fig. 5D). The intersection between the 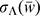 curve and 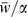 (green or red line for the extreme values of the Poisson's ratio in Fig. 3B) gives the fixed point that is the steady state cell diameter for small γ, see Eq. (9). The slope of 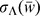 at the intersection gives 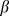, which determines diameter stability, see Fig. 4. We find that the candidate fixed point occurs at very large diameters around 8 µm, within the microtubule unbundling region where 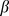
>>1, an unstable case. Had the 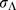 curve in Fig. 5D intersected with the green and red lines at 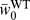 = 3.4 µm, we would have 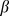
<1 and a stable diameter regulation mechanism. However, the focusing of the microtubules by buckling is too strong to allow for such an intersection for the range of values of *α* found in Fig. 3B, see Fig. 5E. Changing parameters in the model such as reducing the characteristic force for force-dependent microtubule catastrophes by a factor of ten (Fig. 5D), increasing the hydrolysis rate by up to a factor of five (not shown), or increasing the stiffness of microtubules by up to a factor of ten (not shown) did not modify the above conclusions since 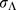 did not change significantly.

The models of Eq. (12) and Fig. 5 are not the only possibilities. Additional microtubule-based mechanisms could interpolate between these two models. For example, if Tea1 delivery happens after some time after first touch of microtubule tip to cell periphery, this would give a larger 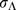 compared to Fig. 5D. Tea1 tip delivery events do seem to be distributed over a wider area compared to Fig. 5D (see experimental data in Fig. 3E in [41]) but the dependence on cell diameter has not been studied. Overall, it appears that several non-trivial mechanisms would have to be added to microtubule geometric alignment model to achieve the desired effect of a signal with an approximately shape-independent width.

One additional mechanism that would enhance stability is that with a growth signal that can develop its own, approximately microtubule-independent width, likely via a reaction-diffusion mechanism [41], [43], [44]. In this case the role of the microtubules would be to provide a target for the growth signal by delivering landmark proteins that direct the signal to the cell tip. Such a mechanism would be consistent with experimental observations that show that the lack of fully functional microtubules or missing polarity proteins delivered along microtubules lead to defective cell shapes that still show polarized growth. For example, cells missing Tea1 can grow a third tip out of the center of the cell [12] and mutations of microtubule-associated protein Alp1 can lead to curved cells [45]. Mutations of cysteine 354 in beta-tubulin changes the overall rate of microtubule growth, shrinkage, catastrophe, and rescue [46]; these changes lead to partially misplaced Tea1 and often to growth from the side of the cell [46]. These cells are also late or defective in initiating bipolar growth, suggesting that the landmarks are necessary to place a new growth site as the cell becomes longer and more mature [46]. Spheroplasts treated with microtubule inhibitor MBC are able to polarize and extend growth projections [16]. Membrane-bound Mod5 appears to cooperate with Tea1 to maintain a robust Tea1 distribution [41]. Related work in budding yeast also supports the ability of the Cdc42 system to break symmetry and establish a polarized growth zone independently of microtubules [43], [47].

In the next section we show how a model with growth zones, microtubules, and landmarks that may be necessary to establish a stable cell diameter can explain several features of cell shape in wild type and mutant cells.

### Model for Shape Maintenance by Growth Zones, Microtubules, and Landmarks

**Figure 6:**
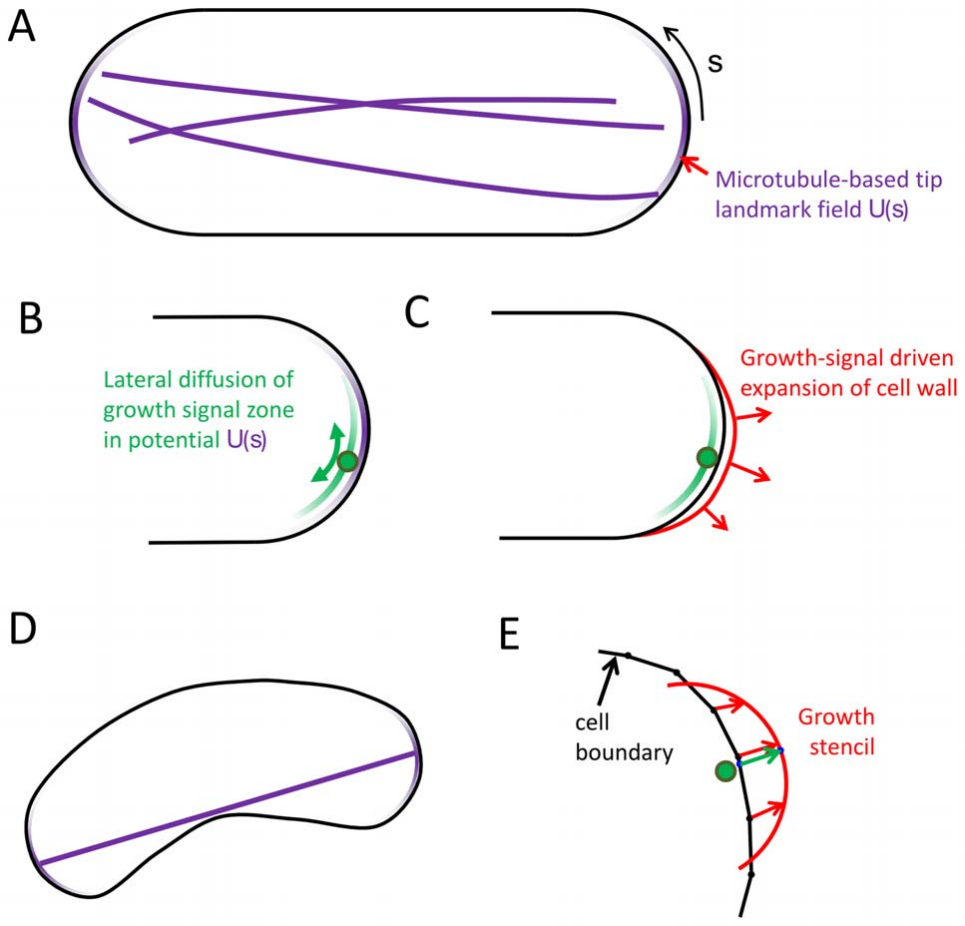
Model with growth zones, microtubules and landmarks (see main text and Methods for detailed description). **A.** Model schematic shows cell outline (black) and the potential *U*(*s*) defined by the microtubule ends at cell tips (purple gradients). **B.** Center of diffusing growth zone (represented by a green circle) moves diffusively in the microtubule-tip-based potential. **C.** Growth signal (green gradient) leads to local cell wall expansion. **D.** A straight line (purple) representing the microtubule system extends towards local length maximum to define the center of the *U*(*s*) potential. **E.** Points on the cell outline move towards the cell stencil (red) centered at the position of the center of the growth signal and oriented normal to the cell contour.

To investigate how the microtubule and tip signal growth components of shape maintenance fit together, we built a qualitative model that includes signal-dependent growth, diffusing growth zones with a native width as from a reaction-diffusion system, and an axis-sensing microtubule system that delivers landmarks to the cell tips (see Fig. 6). Then we explored the parameter space of the model. Here we show that changes to the focusing of the microtubules and the dynamics of the Cdc42 system can lead to bent or bulged shapes, and we describe how many of the known aberrant shapes can be understood within this modeling framework.

In the model we assume that the landmark proteins, such as Tea1, that are delivered by the microtubule system provide an attractive potential *U*(*s*) at cell tips for the center of the Cdc42 growth zone signal (purple zone in Fig. 6A). This potential, arising from the interaction between Tea1, Mod5 [41] and associated proteins on the cell membrane is approximated as a Gaussian distribution of width 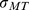 and depth *U*_0_. We assume that the Tea1/Mod5 interactions allow 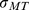  and *U*_0_ to be independent of cell length and diameter. The molecular basis of the interactions between the Tea1 zone and the Cdc42 system have not been established [40], [48], [49]. Recent work has shown that Tea1 and Pom1 mark cell tips by organizing in dynamic clusters of varying density [44], [50] and presumably so does Cdc42. We anticipate that the loose interaction between the Tea1/Mod5 and Cdc42 zones can be captured by a diffusion in a potential process: the constant assembly and disassembly of the Cdc42 clusters in the cap would lead to random motion of the center of the growth signal zone that is biased towards the minimum of the *U*(*s*) potential. This diffusion process can be quantified by one additional parameter, 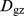, the intrinsic diffusion coefficient of the center of the Cdc42 signal. The standard deviation of the growth signal Λ(*s*) is assumed to be fixed to a value 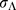, independent of cell shape. An approximately-constant 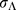 could arise from a Cdc42 reaction-diffusion system and its regulators [43]. Vesicle delivery and removal of Cdc42 can also regulate the size of the Cdc42 zone [47]. We do not write explicit equations for the concentrations in the Cdc42 system because many quantitative and molecular details about those interactions are unknown in fission yeast. However, a known property of the solutions to such equations is that kinetic rates and diffusion coefficients can give rise to a robust length scale and spatial structure that could remain approximately constant as cells double in size [44]. The phenotypes of wider or narrower diameters seen in Cdc42-regulator deletion mutants such as Rga4Δ and Gef1Δ [11] or overexpression of Gef1 [15] would correspond to different 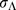 but here we do not model the mechanisms determining 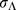.

For simplicity, we study the model in two dimensions. We anticipate that features of the model, such as how far from the tip the growth zone normally diffuses and how often it escapes the microtubule-based potential at the tip, do not depend qualitatively on the dimensionality. (The diffusion of the center of the growth signal along the cell cortex is marginally compact in 2D, similar to 1D compact exploration [51]. Each individual microtubule is also typically found on a 2D plane.) A 3D model could have additional features such as allowing corkscrew-shaped cells that are neglected here. We developed a version of the 3D axisymmetric growth model that could be plugged into the 2D model that uses a protruding stencil for a growth projection from a 2D contour (see Fig. 6 and Methods for details). The stencil size scales to match the width of a growth zone. We used a stencil model since a direct conversion of the model of Fig. 2 to two dimensions does not work because an elastic boundary under turgor pressure in 2D always becomes circular. The growth stencil has the shape of a cross-section of the 3D tip shape derived earlier for a Gaussian growth-factor signal (Fig. 3A). Instead of calculating the shape change based on remodeling under pressure, the outline deforms to accommodate the protrusion of the stencil. The 2D model allows us to examine shapes that would not be axially symmetric without the added complication and computation of a fully three-dimensional model.

Finally, we assume that the microtubule system marks the two most distant parts of the cell that correspond to the two cell tips. This is represented in the model as a line from one point on the cell boundary to another. (This would approximation would fail for extremely bent cell shapes where microtubules cannot extend to the tips and touch the sides [20]–[22]). During every step of the simulation, the line representing the microtubule system repeatedly attempts to increase length by small movements of these two points. This process finds a local maximum of distance between two points on the outline, and for simple shapes such as a rectangle capped on opposite sides by semicircles the process finds the global maximum of distance between points on the entire boundary. The ends define centers of potential *U*(*s*) on the cell boundary for diffusing growth zones.

To explore the family of shapes produced by the above model we started from a 2D cross-section of the cell shape calculated in the 3D model (Fig. 3A) and length 8 µm. This shape was evolved until the long axis of the cell had doubled. For cells that did not elongate linearly, we ended the simulation at three times the time it would take the long axis to double were the cell growing straight. We simulated cells with either one or two growth zones since some shape mutants grow in a monopolar manner (single growing tip, such as *tea1Δ* [12]) while others are bipolar (two Cdc42 zones, such as some *for3Δ* cells [52]).

**Figure 7:**
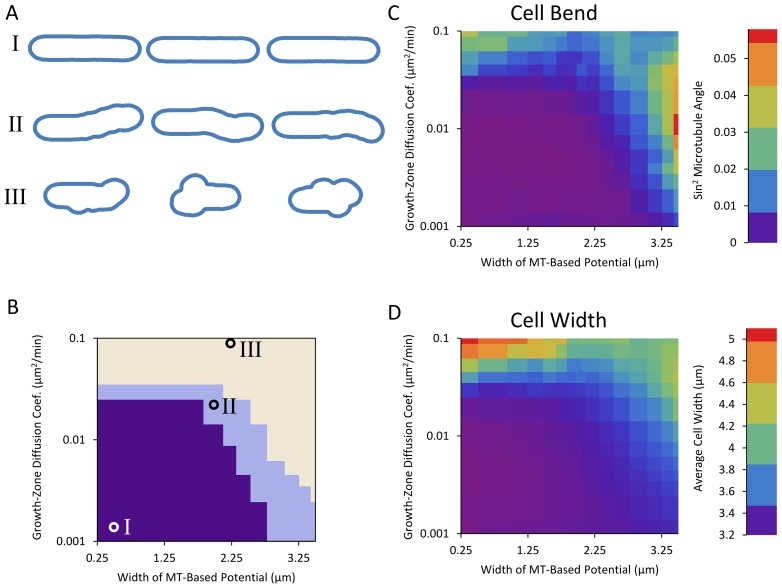
Two-dimensional model with one growing tip generates three families of shapes. **A.** Examples of simulated cell outlines (as described in section ‘Model for Shape Maintenance by Growth Zones, Microtubules, and Landmarks’). Three regions in parameter space show occurrence of: (I) straight cells, (II) bent cells, and (III) wide cells. Cell shapes were generated by starting from an outline of a 8 µm long cell with tips shaped according to the model of Fig. 6 and a growth zone placed at one tip. The model was evolved until cell length doubled or thrice the amount of time necessary for a straight-growing cell to double had elapsed. **B.** Regions of different shapes as function of growth zone diffusion coefficient *D*_gz_ and standard deviation of microtubule-based potential σ_MT_. Circles on plot indicate parameters used for the shapes in panel A. For the definition of the regions, see Methods. The depth of the potential was *U*_0_ = 0.2 µm^2^/min, a value that shows a range of model behaviors. If the potential is very deep, any diffusion coefficient that allows the growth zone to escape from the tip also allows it to explore the side of the cell. If the potential is very shallow, a diffusion coefficient that allows the growth zone to be confined also precludes exploration of most of the cell boundary during the growth phase of the cell. **C.** Cell bend, measured as squared sine of angle between initial and final cell axes as described in Methods as a function of the same parameters as in panel B. **D.** Cell width, measured as described in Methods, as a function of same parameters as in panel B.

**Figure 8:**
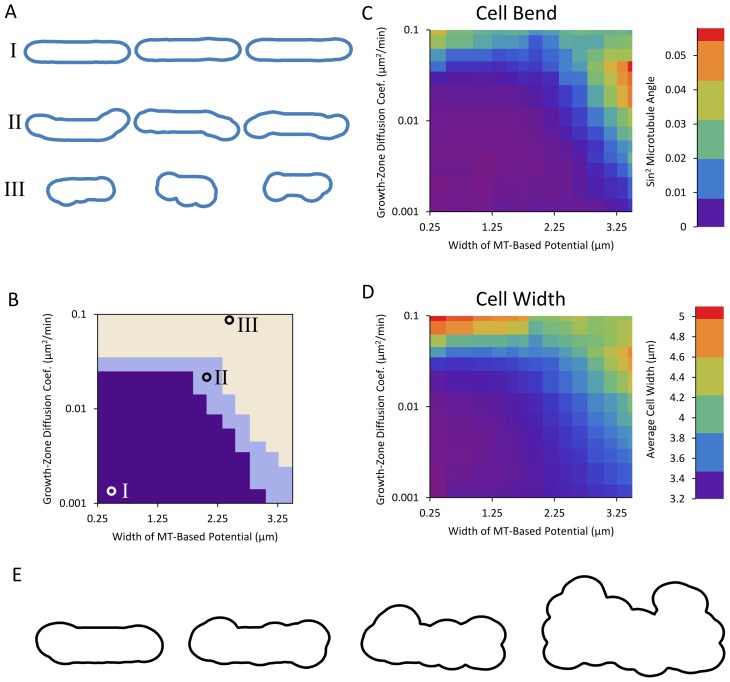
Two-dimensional qualitative model with two growing tips generates three families of shapes. **A–D.** Same as Fig. 7, but with two growing tips. **E.** Evolution of bulged cell (parameters indicated by circle for region III) with two diffusing growth zones at long times. Model evolved for (going right) one, two, three, and ten times the amount of time necessary for a straight-growing cell to double.

Simulations reveal three families of shapes: straight cells, bent cells, and bulged cells (see Figs. 7, 8). For small diffusion coefficients and narrow microtubule-based potentials, cells grow approximately straight (region I on Figs. 7 and 8). As the potential defined by the microtubule becomes wider, if the diffusion coefficient is large enough that the growth zone can move away from the tip during the lifetime of the cell, the cell often grows away from the axis of the cell, resulting in a bent final shape (region II on Figs. 7 and 8). Finally, as the diffusion coefficient becomes large enough that the potential no longer confines the growth zone or the potential becomes so wide that it extends well beyond the cell tips, the growth zones can explore the entire surface of the cell and the cell develops bulges and diameter increases (region III on Figs. 7 and 8). When cells in region III are evolved over long times, they develop an irregular shape, see Fig. 8E (in region III, in the long time limit, each growth zone would generate a protrusion of changing orientation; the average diameter of this protrusion is determined by a balance of *t* ^1/2^ diffusive growth signal spread with linear extension).

Both the bent (region II) and bulged (region III) cell morphologies have been observed by experimentalists, as we will discuss in the remainder of this section. The ban mutants become banana shaped [7] and our results suggest that this could be the result of the combination of wider Tea1 and other landmark protein distribution with a fast diffusing Cdc42 cap. Thus, they may provide an experimental window into the interrelationships among growth, Cdc42 signaling, and the microtubule system. We note that our simulations show equal numbers of S-shaped and banana-shaped cells while prior reports show primarily banana shapes [7]. One possibility is that the model of Fig. 6 is correct in that initial cell bending is due to diffusing growth caps. Aspects of the microtubule system not included in the model might subsequently preferentially stabilize banana shapes as compared to S-shapes: for example, U-shaped buckled microtubules are more likely to occur as compared to S-shapes [42] but the model of Fig. 6 does not account for microtubule buckling. Microtubules in the *ban5-3* mutant tend to be shorter during interphase [7], and the shape of these cells often includes sharp bends. Since the *ban5-3* mutation is on the gene encoding for alpha tubulin Atb2 [53], the resulting cell shape can be attributed to a failure of the microtubule system to reach and indicate the tips for growth, consistent with our model. Another possibility is that microtubule buckling is the primary cause for some of the banana shapes, rather than growth cap diffusion: the landmark distribution generated from buckled microtubules would lead to banana-shaped cells. Images of *ban2-92*, *ban3-2*, and *ban4-81* mutants do show a buckled microtubule bundle on one side of the cell [7] but what is cause and effect is unclear. The mechanism behind shape in these ban mutants might act through components of the microtubule organizing centers attached to the nucleus [54]. We propose experimental measurements of active Cdc42 zone diffusion in the ban mutants to help separate cause and effect in these shape mutants.

Long cells blocked in G2 also become curved in a way similar to the banana mutants [7], . This could be related to a cell-length limit for the normal response of the microtubule system: if mictrotubules are unable to extend all the way to cell tip, this might give rise to a wider and/or shallower potential *U*(*s*).

Bulge-shaped cells have also been observed in Mid1 mutants [28], [56] and in the *sla2Δ* cells shown in Fig. 1H [57]. We suggest these shapes might be related to a rapidly diffusing growth zone in combination with defects in microtubule organization. Alternatively, unregulated random nucleation, growth and disassembly of Cdc42 growth zones (as occurs during mating [58]) could explain these shape mutants. To date, bulged mutants have not been explored as much as other aberrant shape phenotypes, possibly because such a trait may be caused by factors other than mutations.

Here we did not address T-shaped cells [12]. These phenotypes may occur because of a failure to initially place the growth zone at the tips.

## Discussion

### Summary of This Work

This work addresses three questions: (1) Can a physical model for how fission-yeast cell shape could depend on a cortical signal reproduce the observed cell diameter and tip shape using the measured active Cdc42 profile? (2) What are the ramifications of a shape-dependent signal for growth, and can a mechanism where the width of the tip growth signal is determined by microtubule focusing lead to stable regulation of diameter? (3) Can a number of abnormal fission yeast shapes be understood in terms of disruptions to a few interacting modular components that link the cytoskeleton to Rho GTPase signaling?

To address the first question (1), we developed a coarse-grained mathematical description of the cell boundary as an elastic shell shaped by turgor pressure (Fig. 2A), and of how the shape of this boundary would change due to continuous renewal of the boundary material (Fig. 2B). Results from this model include a rate of signal width to cell diameter in accord with experimental results [11], [15] (Fig. 3A). We predict this ratio remains the same in diameter mutants that accumulate a Gaussian Cdc42 distribution at cell tips. We also predict how cell diameter equilibrates after a sudden changes to growth signal (Fig. 3D).

To address the second question (2), we give an account of how feedback between a growth signal and cell shape might affect diameter. Results from this model include a condition for stable diameter regulation (*β*<1, Fig. 4), and data from a detailed microtubule simulation ([42], Fig. 5) suggesting that simple feedback between growth signal and cell shape through physical exploration by the microtubules may not be sufficient to establish a constant cell diameter. We predict that in mutant cells that become wider or thinner over successive generations, as in Fig. 4C, the growth signal width increases rapidly with increasing cell diameter, such that *β*>1.

To address the third question (3), we describe a qualitative model that incorporates components of fission-yeast shape regulation to provide a basis for understanding shape abnormalities (Fig. 6). The model includes coarse grained versions of the microtubule-dependent tip-sensing mechanism, the landmark proteins delivered by motor proteins along those microtubules, and the active-Cdc42-dependent growth described in the first part (1). The results of Fig. 7 and 8 showing an exploration of model parameters predict correlations among cell shape defects, distribution of microtubule-dependent landmark proteins, and mobility of Cdc42 growth zones.

As a whole, this theoretical work describes a framework for understanding how shape is regulated and maintained in fission yeast, and motivates experimental investigation into the physical components of the cell that correspond to mechanisms of the model.

### Comparison to Other Models for Tip Cell Wall Growth

Our model of signal-dependent growth relates to previous models of tip shape in other cell types. We note that there are some differences between fission yeast and other tip-growing cell types. Pollen tubes, for example, secrete pectic polymers to build their cell wall at the cell tip and they are cleaved of methyl groups as they mature [59], [60] while fission yeast lacks pectin and its cell wall contains primarily glucan and chitin [61], [62]. Vegetative fission yeast also does not have a Spitzenkörper [1], [63], the organizing center for vesicle delivery in growing tips of fungal hyphae, so our model differs from models that have investigated the consequences of Spitzenkörper-dependent tip growth [64], [65].

Several models for plant and hyphae tip growth that rely on gradients of mechanical and viscous properties of the cell wall along the cell tip [25], [60], [64], [66], [67]. Here we assumed a uniform Young's modulus, *E*. We are not aware of measurements of the modulus as a function of distance from tip, *E*(*s*) in fission yeast. Since the expansion rate in our model depends on the ratio Λ(*s*)/*E*, such a dependency could be mathematically folded in to Λ(*s*), with the new signal being Λ(*s*)/*E*(*s*). This implies that softer parts of the cell wall extend faster under the same stress and signal. This effect could be relevant for cell growth immediately after septation when the new and old ends may have different mechanical properties: in budding yeast the scar region that contain septins has a 10-fold larger modulus compared other parts of the cell [68]. However Λ and E are not independent parameters so if future experimental measurements show an *s*-dependent modulus, they would point to additional complexities not included in our model that would require modeling of cell-wall renewal at a molecular-level.

We have also neglected passive plastic cell wall flow (flows in our model are due to cell wall remodeling). We believe this is a good approximation for fission yeast cells since they stop growing from the tips when entering mitosis, without bursting or obvious cell wall thinning. Deformed fission yeast cells quickly recover their shape after undergoing large deformations, also indicative of elastic behavior [20]. We note that the relative importance of passive plastic deformation versus biochemical-driven expansion are difficult to disentangle, as has been discussed extensively in plant cell growth [69], [70]. Our work motivates further investigation of this issue in fission yeast.

Our model is most closely related to Dumais et al. [25] who modeled the cell wall of tip-growing plant cells (such as elongating root hair cells of *M. truncatula*) as a thin viscoplastic shell. In Dumais et al., the mechanical properties of the wall—extensibility, yield stress, and Poisson's ratio—vary with distance from the tip and their interaction gives rise to shape. The extensibility function plays a similar role to our Λ(*s*) and both models share the same algebraic expressions for elastic shells [26]. While the equations that describe the steady state are mathematically very similar, in Dumais et al., the delivery profile of the cell wall material is assumed to be tuned according to cell wall expansion, to maintain a constant wall thickness. We assume that the delivery profile, proportional to Λ(*s*), guides local cell expansion by directing local exocytosis and cell wall remodeling enzymes to specific parts of the cell. Since we model the cell wall as an elastic material, the cell wall would not flow during a transient when delivery is stalled, unlike in Dumais et al. We also make the assumption that the material delivered is able to maintain a wall of constant thickness, consistent with recent theoretical work in bacterial cell wall remodeling [71]. We also assume a different expansion rate: in Eq. (3) we assume the expansion rate is proportional to local strain while Dumais et al. assume that the strain rate (corresponding to our ξ) is distributed according to a local energy minimization.

Campas and Mahadevan [67] identify two length scales in tip growing cells, one describing the distance away from the tip at which the polymers of the cell wall become increasingly cross-linked causing a transition from a fluid to a solid wall (in pollen tubes this depends on pectin methyl-esterases and their inhibitors) and another describing the distance from the tip where the rate of material deposited to the wall falls off. These two length scales lead to a spectrum of possible cells diameters and shapes where the radius of curvature at the tip and the radius of the cell body differ. Here we assume cell wall expansion is linked to delivery of wall material through Λ(s). Thus, changes of tip shape and diameter rely on different Λ(s) profiles.

Fayant et al. [60] developed a finite element model for growing lily pollen tubes and use the model alongside experiments to identify a cell-wall component responsible for changing the mechanical properties of the wall. In that model, the Young's modulus varies with the angle between the tip and the long axis of the cell, reflecting a continuous maturation process as wall material moves backward from the tip. Experiments reveal this maturation process to be the esterification of pectin. They assume exocytosis acts to maintain constant cell-wall thickness during expansion.

The rod shape is not exclusive to eukaryotes: a few well-studied bacteria maintain a shape that is similar to that of fission yeast with growth occurring along the cylindrical cell body or at cell tips, depending on the organism [72]. *B. subtilis*, *E. coli*, and *C. crescentus* grow by patterned insertion of peptidoglycans into the sidewall using a MreB-dependent mechanism and some disagreement remains as to whether this operates by circumferential motion of a complex including MreB [73], [74] or as a consequence of a helical MreB structure [75], [76]. Huang, Wingreen and others used molecular-level models to describe the growth of Gram-negative bacteria such as *E. coli* [75], [77], [78]. In these studies, an elastic network glycan strands and peptide crosslinks expands as material is inserted with some orientation preference. These models capture cracked-cell shapes that result from patches of defects in the network [77]. Jiang and others also put the growth and shape of multiple Gram-negative cells into a common framework [79]. They use a continuum model of the peptoglycan network to show how growth, cell-wall mechanics, and the bacterial cytoskeleton can interact to produce shape. According to that study, a dynamic helical bundle of MreB exerts forces on the cell wall as it is remodeled, keeping it from swelling in response to the turgor pressure. They use the model to explain shape change after the loss of the MreB helix due to drug treatment. Because the models of bacterial shape by Huang et al. [77] and Jiang et al. [79] describe cells that use a different mechanism to maintain shape, the pattern of growth is very different from the model of fission yeast described in this paper. However, the concept of remodeling part of the wall, of breaking down a peptoglycan network and inserting new material as in Huang et al [77] is similar to the assumptions of our model, even if the region that expands is different.

### Modular Control of Fission Yeast Shape

Shape regulation, as described in the last part of this work (Fig. 6), is essentially modular. The separate components—the microtubule system, the Cdc42 signaling, and the landmarks—interact but are described by separate genes and consist of separate proteins. And to some extent they can operate separately: many of the shaping mechanisms are understood because the other modules continue to work if they are disrupted, as in the case of banana-shaped cells where the Cdc42 cap may function normally but the landmarks are misplaced. Our description of the bent cell shaping mechanism differs from the ideas presented in literature that suggest that the banana shape comes from a length scale within a reaction-diffusion equation [55] (note: such a mechanism also does not distinguish between S and banana shapes) or from whole-cell buckling [80]; these contrasting explanations motivate further experimental study of the ban mutants.

The framework described in this work also appears to be consistent with recent observations of spheroplasts, cells that have become round because the wall has been enzymatically digested [16]. Despite their round shape, these cells form a growth zone of the proper size (and of the altered size in cells missing components of the Cdc42 system) at a random location. Because the growth zone size in this case seems to be independent of the physical shape of the cell, this argues that the Cdc42 system has an intrinsic length scale that ultimately sets the diameter of the cell. The fact that tip growth can occur in spheroplasts treated with microtubule inhibitors [16] provides further support for the conclusions of this work. The fact that spheroplasts with microtubules unperturbed by drugs grow a straighter protuberance [16] is also consistent with the picture advanced here. This particular study also implies another aspect of shape regulation, recovery: cells can recover polarized growth from a spherical shape. This indicates that the machinery is robust enough to reestablish polarity even in cases where it is lost completely.

How would oscillations and fluctuations [15] affect our results? Since the fluctuations and oscillations of Cdc42 take place over a period of around 5 minutes, we expect that the growth of the cell (which doubles over hours) evolves according the time-averaged tip Cdc42 tip signal. Cdc42 oscillations and fluctuations may enhance the mobility of the Cdc42 zone by increasing parameter *D*_gz_ in Fig. 6B. Oscillations and fluctuations could play a bigger role in the establishment of multiple growth zones, change of polarity, and in the efficient use of resources (which may be concentrated at one tip in small cells that only have enough growth machinery to grow at one tip) [15]. Here we showed that certain predicted aspects of calculated shape and parameter dependence of the model are not strongly dependent on whether one or two growth zones is used (see Fig. 7, 8).

In addition to genetic and pharmacological manipulation, prior studies on fission-yeast shape also included perturbations to the physical environment of the cell. In particular, in two prior studies cells were confined within curved chambers to study the response of the growth machinery [20], [21]. One study used elastic microchambers [20], the other curved passages [21]—but in both cases they found that cells forced to adopt curved morphologies misplaced landmarks due to a change in the organization of the microtubule system. Both of these studies support the framework of fission-yeast shape regulation proposed in this work (though in Fig. 6 we assume the microtubules can mark the most distant part of the cell and do not account for extremely bent cells). Terenna et al. [21] found that Tea1 and Bud6 get more tightly focused at the cell tips upon confinement of banana-shaped mto1Δ cells into straight channels. While we do not offer an explanation for this observation, our study motivates similar studies of the response of Tea1 and Cdc42 upon confinement of wild type and shape mutants to channels of varying diameter. Such experiments may allow measuring the value of parameter *β* that we propose is important for determining cell diameter.

Studies of the shmoo shapes in mating yeast could offer possibilities to test some of our predictions. Since pheromone concentration influences Cdc42 dynamics [58], use of artificial pheromone gradients may allow shaping of the Cdc42 profile to observe the resulting change of shmoo shape. Use of electric fields to control Cdc42 distribution [81] is yet another approach.

Finally, we have identified three mechanisms that could lead to round cells: (1) Establishment of a very wide Cdc42 region leading to a diameter comparable to cell length; (2) Sensitivity of cell growth signal to cell diameter (case *β*>1); (3) Highly motile Cdc42 patch (the latter leading mostly to bulgy cells). Case 3 is similar to the random motion of a Cdc42 parch along the cell periphery in mating cells [58]. Future studies imaging the distribution and dynamics of Cdc42 and the cytoskeleton in wild type and mutant cells could help distinguish among these possibilities and test the validity of the proposed modular mechanism.

## Methods

### 1. Methods Related to Model for Remodeling under Turgor Pressure (Fig. 2)

#### Evolution of tip shape as function of growth-factor signal Λ(s)

The differential equations described by Eq. (4) can be rearranged to give a differential equation for *v*_t_ (as in [25]):
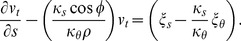
(14)The geometric relations 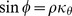 (see Fig. 1) and 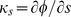 allow this to be simplified:
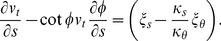
(15)The left-hand side is 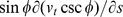, so the expression can be written:
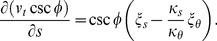
(16)Adding the boundary condition 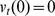, which is imposed by axisymmetry, the system admits the following solution:
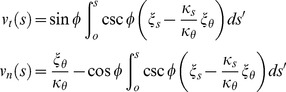
(17)
Eq. (5) of the main text is obtain by substituting Eq. (3) into Eq. (17), substituting Eq. (2) followed by (1) into the result. Eq. (5) shows that the growth velocity depends on the ratio *G_max_ P/Eδ*,

The axisymmetric nature of the model allows the cell surface to be represented by a tip-to-tip contour. For calculation purposes, we discretized the contour to a series of points, with *s* being the sum of segment lengths from tip to point. Derivatives and curvatures were calculated using the five-point stencil. For a given cell shape and *Λ*(*s*), numerical integration of Eq. (17) gives the normal and tangential velocities. Every point on the contour was moved by 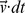 (where 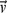 is the total velocity vector). After this step, new point positions along the segmented contour were calculated to maintain equal separation between the points, with additional points added as the contour becomes longer to maintain approximately the initial spacing. We checked that *dt* and the spacing between points along *s* were sufficiently small for numerical integration. To calculate whole-cell shapes in Fig. 3A, steady-state tip shapes were joined to a cylindrical middle section and the length of that section was chosen to give constant volume.

### 2. Methods Related to Model for Shape Maintenance by Growth Zones, Landmarks, and Microtubules (Fig. 6)

#### Cell boundary

The outline of the cell border is modeled as a series of discrete points (see Fig. 6E) as described in Methods section 1 for the axisymmetric growth model. Here Catmull–Romm splines were used for the interpolation during contour resegmentation. This alleviates an effect where repeated linear resegmentation erodes the contour, especially as *dt* becomes small.

#### Discretization of the cell boundary

For the simulation to accurately represent the continuum model, the number of points along the cell boundary should be chosen so that the distance between points is much smaller than the inverse of any curvature along the contour representing the cell outline. Therefore the initial number of beads *n* is chosen by:
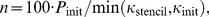
(18)where *P*_init_ is the length of the perimeter at initialization, *κ*_stencil_ is the curvature at the tip of the stencil, and *κ*_init_ is the curvature at the tip at initialization.

#### Growth stencil (Fig. 6E)

For the model of Fig. 6, we import a tip outline from the three-dimensional model. This is defined as the intersection of the three-dimensional outline with any plane that includes the axis of symmetry of the three-dimensional outline, trimmed back at the section of the outline where the cell becomes cylindrical. A Gaussian growth-factor profile was used to generate this outline, which can then be scaled to match the width parameter of a growth zone. The axis of symmetry of this stencil is then aligned to the normal vector at its position on the contour representing the cell outline. For growth, the tip is moved along this normal vector by *v dt*, where *v* is the magnitude of the growth velocity vector, and points along the contour representing the cell outline are moved towards points that are the same distance along the stencil. Points on the contour representing the cell outline do not move if that movement would be inward (if the inner product of the normal vector with the direction to the corresponding point on the stencil is negative), and only move a maximum distance of 2 *v dt* (1−*s/S*) where *s* is the distance from the growth zone and *S* is the maximum distance along the growth stencil. This prevents discontinuities in the contour representing the cell outline.

#### Diffusing growth zone (Fig. 6B)

While the cells expands, the center of the growth signal zone diffuses in a one-dimensional potential, *U*(*s*), or equivalently 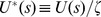, where ζ is the drag coefficient. Potential wells surround the tips of the microtubule, and they have the form of a Gaussian with standard deviation σ_MT_ and depth *U*_0_. The movement of the growth zone is simulated according to Brownian dynamics:
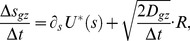
(19)where *s*_gz_ is the growth-zone position, *D*_gz_ is the growth-zone diffusion coefficient, *R* is a random number picked from a Gaussian probability density function with standard deviation one and mean zero, and *Δt* is the integration time step.

#### Choice of integration time step Δt

Because, for each time step, only the local gradient of the potential, ∂_s_ *U* ^*^(s), is used to describe the interaction of the growth zone with the potential, the distance that the growth zone travels during one time step due to both that interaction and diffusion should be small compared to the scale of the features of that potential. After excluding numerical prefactors of order unity, this leads to the conditions:
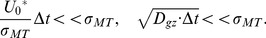
(20)Changes to the contour representing the cell outline should also be small compared to the features of that outline during any single time step. An appropriate scale for the features of the outline is the inverse of the stencil-tip curvature, 1/κ_stencil_, and changes to the outline go as *v*_growth_ Δt. This gives the additional condition:
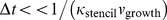
(21)To meet the above criteria we chose the time step to be:
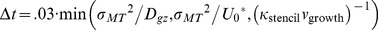
(22)


#### Measuring bend and width from cell outlines

Because the cell shapes tend to be mostly tubular (visual observation) the degree of bend is approximated by the angle between the line representing the microtubules, which has two ends that move in short steps when it will increase length (described above in ‘Model for Shape Maintenance by Growth Zones, Microtubules, and Landmarks’), and the initial cell axis (*i.e.*, the angle between the purple line and the horizontal in Fig. 6D). We used the squared sine of this angle as a metric with a non-zero average. This measure has some limitations; for instance, a simulated cell growing at both tips that develops a c-shape will appear to have no bend. However, conditions leading to c-shapes also lead to other bent shapes cells and the squared sine is a representative measure. Measuring width also relies on the line simulating microtubules for detecting the endpoints. Moving away from each tip, the distances between pairs of points are compared out to half the cell length away from the tip and the maximal distance is considered to be the width. To divide cells into the three categories of straight cells, bent cells, and bulged cells (Figs. 7 and 8), we chose thresholds for the degree of bend (squared sine of 0.0015) and measured width (3.45 µm). Category I, straight cells, included only regions of parameter space where both the degree of bend and the measured width were below the threshold. Category II, bent cells, included only regions where the degree of bend was above the threshold and the measured width was below the threshold. Category III included everything else. These thresholds were set by trial and error to match what by inspection appeared to be the three categories.

## Supporting Information

Table S1 Parameters used in this paper.
(DOCX)

Table S2 Parameters for the two-dimensional model of Foethke *et al* (see Fig. 5). For the units, distances are given in µm, forces are given in pN, and times are expressed in seconds. Bolded values are changed for some simulations as described in the text. These values are used in a configuration file for the Cytosim program found at http://www.cytosim.org/cytosim/index.html. We used the compiled version 3.0 beta found on that site, which comes with a set of configuration files. The default values for the microtubule simulation can be found in the pombe.cym file.
(DOCX)
