# Supplementary material for: Model of Fission Yeast Cell Shape Driven by Membrane-Bound Growth Factors and the Cytoskeleton
Source: PLoS Comput Biol. 2013 Oct 17;9(10):e1003287. doi: 10.1371/journal.pcbi.1003287 (PMC3798282; doi:10.1371/journal.pcbi.1003287)
Supplement: Table S1 — Parameters used in this paper. (DOCX) [file pcbi.1003287.s001.docx]

**Table S1.**

| Parameter | Value | Source Reference |
| --- | --- | --- |
| *w*, cell diameter | 1.6 μm | [42] |
| *P,* turgor pressure | .85 MPa | [22] |
| δ, cell-wall thickness | 200 nm | [31] |
| *E,* Young’s modulus of cell wall | 101 MPa | [22] |
| *L*_0_, cell length at birth | 7.5 μm | [24] |
