# Supplementary material for: Model of Fission Yeast Cell Shape Driven by Membrane-Bound Growth Factors and the Cytoskeleton
Source: PLoS Comput Biol. 2013 Oct 17;9(10):e1003287. doi: 10.1371/journal.pcbi.1003287 (PMC3798282; doi:10.1371/journal.pcbi.1003287)
Supplement: Table S2 — Parameters for the two-dimensional model of Foethke et al (see Fig. 5). For the units, distances are given in µm, forces are given in pN, and times are expressed in seconds. Bolded values are changed for some simulations as described in the text. These values are used in a configuration file for the Cytosim program found at http://www.cytosim.org/cytosim/index.html. We used the compiled version 3.0 beta found on that site, which comes with a set of configuration files. The default values for the microtubule simulation can be found in the pombe.cym file. (DOCX) [file pcbi.1003287.s002.docx]

**Table S2.**

| Container | Parameter | Description | Value |
| --- | --- | --- | --- |
| simul | time_step | time step for integration | 0.01 |
| simul | kT | temperature in energy units | 0.0042 |
| simul | viscosity | viscosity of cytoplasm partially determines mobility of nucleus | 0.9 |
| simul | precondition |  | 0 |
| simul | binary_output |  | 0 |
| space pombe | geometry | shape of the cell boundary; ‘capsule’ indicates spherocylinder; 2.4 is half the cylinder height; 1.6 is the radius of the hemispheres | **( capsule 2.4 1.6 )**  **adjusted to change dimensions of cell, see Figure 5** |
| fiber microtubule | rigidity | modulus for bending elasticity | **30 (up to 300)** |
| fiber microtubule | confine | sets forces between fiber and confining space | inside, 200 |
| fiber microtubule | activity |  | dynamic |
| fiber microtubule | unit_length | length of discrete units of assembly/disassembly | 0.008 |
| fiber microtubule | growing_speed | speed of assembly | 0.06 |
| fiber microtubule | shrinking_speed | speed of disassembly | -0.15 |
| fiber microtubule | hydrolysis_rate | hydrolysis rate of g-units, from which the catastrophe rate is calculated | **0.058 (up to 0.29)** |
| fiber microtubule | growing_force | characteristic force of polymer assembly, used for force-dependent catastrophes | **1.7 (down to 0.17)** |
| fiber microtubule | shrinking_fate | what happens when a microtubule reaches the minimum authorized length (below) | rescue |
| fiber microtubule | min_length | minimum authorized length | 0.5 |
| sphere envelope | point_mobility | mobility of points on the surface | 0.05 |
| sphere envelope | radius | the radius of the sphere | 1.3 |
| sphere envelope | confine | flag to confine this object | inside, 200 |
| sphere envelope | piston_effect | if true, use special formula to calculate mobility (presumably as in Foethke, et al.) | 1 |
| bundle microtubule_bundle | fibers | the name of the fiber in the bundle | microtubule |
| bundle microtubule_bundle | nb_fibers | the number of fibers in the bundle | 4 |
| bundle microtubule_bundle | overlap | the length of the zone where the fibers in the bundle overlap | 0.5 |
| bundle microtubule_bundle | stiffness | stiffness of the links that connect overlapping fibers | 1000 |
| nucleus nucleus | sphere | name of the sphere | envelope |
| nucleus nucleus | nb_bundles | number of MTOCs | 4 |
| nucleus nucleus | bundles | the bundles at the MTOCs | microtubule_bundle |
| nucleus nucleus | stiffness | stiffness of assembly links | 200 |
